# Discovering Brain Mechanisms Using Network Analysis and Causal Modeling

**DOI:** 10.1007/s11023-017-9447-0

**Published:** 2017-10-25

**Authors:** Matteo Colombo, Naftali Weinberger

**Affiliations:** Tilburg Center for Logic, Ethics and Philosophy of Science, Tilburg University, P.O. Box 90153, 5000 LE Tilburg, The Netherlands

**Keywords:** Causal inference, Brain connectivity, Dynamic causal models, Graphical causal models, Long term potentiation

## Abstract

Mechanist philosophers have examined several strategies scientists use for discovering causal mechanisms in neuroscience. Findings about the anatomical organization of the brain play a central role in several such strategies. Little attention has been paid, however, to the use of network analysis and causal modeling techniques for mechanism discovery. In particular, mechanist philosophers have not explored whether and how these strategies incorporate information about the anatomical organization of the brain. This paper clarifies these issues in the light of the distinction between structural, functional and effective connectivity. Specifically, we examine two quantitative strategies currently used for causal discovery from functional neuroimaging data: dynamic causal modeling and probabilistic graphical modeling. We show that dynamic causal modeling uses findings about the brain’s anatomical organization to improve the statistical estimation of parameters in an already specified causal model of the target brain mechanism. Probabilistic graphical modeling, in contrast, makes no appeal to the brain’s anatomical organization, but lays bare the conditions under which correlational data suffice to license reliable inferences about the causal organization of a target brain mechanism. The question of whether findings about the anatomical organization of the brain can and should constrain the inference of causal networks remains open, but we show how the tools supplied by graphical modeling methods help to address it.

## Introduction

The Human Connectome Project aims to map the neural pathways that underlie human brain function (van Essen et al. 2013). The purpose of this ongoing multi-million dollar project is to acquire and share data about the anatomical connections among brain components, with the goal of producing a detailed *connectome*, or “wiring diagram”, of the human brain (Sporns et al. 2005).

Without such wiring diagrams—it has been claimed—scientists cannot fully understand how causal interactions in the brain produce brain functions, cognitive capacities and behavior (Sporns et al. 2005: 249; Sporns 2012; Bargmann and Marder 2013; Park and Friston 2013). Some have gone so far as to assert that knowledge of the full connectome will reveal how a complex, multi-scale, network system like the human brain “makes us who we are” (Seung 2012).

Knowledge of anatomical connections does help neurophysiologists design experiments, especially on simple invertebrate organisms like the roundworm *C. Elegans* (White et al. 1976; Chalfie et al. 1985; Marder and Bucher 2007; see also Ankeny 2000). Yet, questions remain regarding what exactly a detailed mapping of brain connectivity contributes to the understanding of brain function (Jabr 2012).

A complete connectome will not suffice to understand how the brain works. As Carandini (2012: 509) observes, “we have long known the full connectome for the worm *C. elegans*, detailing the more than 7000 connections between its 302 neurons, and yet we are hardly in a position to predict its behavior, let alone the way that this behavior is modified by learning”. While a complete connectome does not by itself reveal which connections are explanatorily relevant to which behaviors, it may reveal global properties of connectivity, which may in turn constrain mechanistic explanation of behaviour (see e.g., Craver 2016). But the nature of these constraints needs to be made more precise.

Providing a detailed anatomical mapping of the brain is one of several ways that one might represent patterns of connectivity in the brain. One may also represent the correlations among the activities in different components, or the causal relationships by which the activity in one component influences that in another. Across these different representations of connectivity, there is no invariant property that can be captured anatomically (Friston 2011: 16). The relationship between anatomical structure, correlations among activities in distinct brain components, and causal structure is multifarious and complicated (Horwitz 2003; Sporns 2007: 4.3). In what follows, one of our aims is to clarify this relationship, situating our discussion in the broader philosophical literature on mechanism discovery.

There presently exists a robust literature on the use of anatomical information for mechanism discovery (Bechtel and Richardson 1993; Craver and Darden 2001, 2013) and a nascent literature on the explanatory role of network topology (Huneman 2010; Colombo 2013; Levy and Bechtel 2013; Rathkopf 2015; Craver 2016; Kostic 2016). Our present focus is on the use of anatomical information for the discovery of causal networks.

Given that a connectome of the brain is comprised of large sets of nodes, the process of determining which brain components are causally connected presents challenges that are not present in simpler systems. Of course, even a full causal mapping of the brain might fail to provide a full mechanistic explanation for a phenomenon, since it may remain unclear which causal relationships are constitutively relevant to performing a particular function. Nevertheless, it is uncontroversial that mechanisms explain in part through the causal contributions of constitutively relevant components. Accordingly, the use of networks for modeling the causal relationships among brain components merits discussion independently of whether networks provide a distinctive non-causal form of explanation.

One suggestion about the role of anatomical information in mechanism discovery is that models of neural wiring constrain the set of plausible hypotheses regarding the causal organization of target mechanisms. Yet, linking neuroanatomy with the causal organization of neural mechanisms is surprisingly challenging, as is evident from the fact that the best contemporary causal modeling methods in neuroscience hardly rely on available information about anatomical connections (Ramsey et al. 2010: 1545; Friston 2011: 16).

There are two distinctive features of our approach that we take to be crucial for making progress in identifying the most effective strategies for the discovery of brain mechanisms. First, we formulate the question of the relationship between anatomical, functional, and causal connectivity in terms of networks. This corresponds to already existing distinctions in the literature on connectivity in network neuroscience (Sporns 2007) and allows us to formulate more precise questions about the products of different methods for generating and evaluating hypotheses about a mechanism. Second, and relatedly, we distinguish between different ways that anatomical knowledge might contribute to discovery of a mechanism’s causal organization. Such knowledge evidently plays a role in the selection of variables that might pick out constitutively relevant parts of a mechanism, and in designing experiments for learning how a mechanism works (Craver and Darden 2013). Here we are concerned with whether it plays any distinctive role in current, quantitative approaches for causal discovery from brain imaging data.

Our contribution is organized in three sections. In Sect. 2, we regiment our discussion by introducing the basics of network neuroscience. In particular, we distinguish three modes of connectivity in the brain, which haven’t been neatly distinguished in the philosophy of network neuroscience so far: structural, functional, and effective connectivity. We use these concepts to ask the question: How does information about *structural* connectivity contribute to inferences about *effective* connectivity?

In Sect. 3, we examine one strategy for mechanism discovery, which is often employed in neuroscience and that highlights that findings about the anatomical organization of brain circuits can constrain the space of possible mechanisms for a given phenomenon (Craver and Darden 2001, 2013). To substantiate this idea, mechanist philosophers have focused on the discovery of the mechanism of long-term potentiation (LTP) (Darden 2006: Ch 2; Craver 2007: Ch. 7.3). On the basis of the same case study (i.e., Bliss and Lømo 1970, 1973), we provide a more nuanced account of how knowledge of the anatomical organization of the hippocampus facilitated the discovery of the causal organization of a mechanism that might underlie LTP.

As Darden notes, the process of mechanism discovery is an “extended, piecemeal process with hypotheses undergoing iterative refinement”, which may involve several different strategies that do not necessarily generalize to all cases of mechanism discovery (Darden 2006: 272). In fact, the idea that knowledge of anatomical organization guides and constrains inferences about mechanisms for a given phenomenon does not generalize to current causal modeling practice in neuroimaging, where there is “remarkably little evidence that quantitative structural information about connections helps with inferences about effective connectivity” (Friston 2011: 16).

In Sect. 4, we turn our attention to two contemporary quantitative approaches to causal discovery from functional neuroimaging data: dynamic causal modeling and probabilistic graphical modeling. In the hope of bridging the qualitative accounts of mechanistic discovery and quantitative theories of causal discovery from machine learning and network neuroscience, we explain the differing role played by anatomical knowledge in these two approaches. While advocates of dynamic causal models have emphasized their ability to incorporate anatomical information, in practice this information has been used to improve the statistical estimation of parameters in an already specified causal model. Probabilistic graphical modeling, in contrast, makes no appeal to anatomy, but lays bare the conditions under which knowledge of functional connectivity suffices to license reliable causal inferences. In a conclusion, we summarize our novel contributions to the literature on mechanistic explanation and discovery.

## Networks of the Brain and Their Modes of Connectivity

The brain is a complex system composed of intricately interconnected, causally interacting elements. The human brain is composed of blood vessels, glial cells, and neurons with diverse morphology and physiological properties. It includes about 100 billion neurons, each one of which has 10,000–100,000 synaptic connections to other neurons for a total neural wiring length of about 100,000 km. Neurons are organized into circuits and populations. Neural circuits and populations constitute regions and pathways. Development and experience shape the structural and causal organization of these elements, which are responsible for cognitive capacities and behavior.

Network science studies the architectural and topological organization of brain networks, aiming to explain how this organization is responsible for cognition and behavior (Sporns 2011). Graph theory is the primary analytical toolbox used in network science. Graphs are sets of nodes and edges, which allow one to represent complex network systems. Nodes represent elements or components of the system. Edges represent connections between pairs of nodes. Edges can be directed or undirected, and they can be binary (i.e., they can be present or absent) or weighted (i.e., they can take on fractional values). Nodes can be connected directly by single edges or indirectly by intermediate nodes and edges.

If we consider a graphical representation of a brain network, network nodes can represent neural elements such as cells, populations of neurons, or cortical and subcortical regions. Network edges can represent structural connections between nodes such as synapses or axonal pathways. Connections between brain components can then be studied at different spatial and temporal scales, with different instruments, and on the basis of different data.

The choice of nodes and edges is non-trivial. It depends on several factors, including the temporal and spatial scale at which scientists want to study a target network system, on the type of data they can collect, and on their background knowledge about the system. For neuroimaging data, for example, network nodes typically correspond to individual voxels or to aggregates of voxels, which are assumed to pick out portions of different anatomical brain regions of interest; edges typically correspond to the presence of probabilistic associations among the activities of distinct aggregates of voxels, which are inferred from time-series data from brain scans of several experimental participants.

Network measures include the *degree*, *strength*, and *centrality* of a node. The degree of a given node is the number of connections that link the node to the rest of the network. Nodes with high strength make strong connections—where strength is equal to the sum of connection weights. The minimum number of edges that must be traveled to go from one node to the other defines the path length between two nodes. The centrality of a node measures how many shortest paths between other parts of the network pass through that node. Nodes with high degree and high centrality are called ‘hubs’. A *module* is a community of nodes that show a greater number of mutual connection within the community, and fewer connections with nodes of other communities. Because these measures refer to the topology of the network, two nodes of a network can be physically distant but topologically close.

Networks with *small*-*world* topologies have high levels of clustering around hubs and short path lengths (Watts and Strogatz 1998). These networks are “scale-free”, which means that they have the same topological relationships when considered at finer- and coarser-levels of grain (Barabási and Albert 1999). Consider, for example, a graph in which the nodes are cities and the edges indicate which cities may be directly reached via which others. A map of the United States will reveal that certain cities are air-travel hubs and that most cities have comparatively few connections. If one were to zoom into a certain geographical region and also consider the train connections between cities, one discovers a similar pattern of clustering at this level. Small-world networks do not have a characteristic scale, and are useful for modeling systems with multiple-levels of organization like a country or the brain (Sporns 2011: 170–171).

An important distinction in the study of brain connectivity is that between structural, functional, and effective connectivity (Sporns 2007). *Structural connectivity* refers to the pattern of physical or anatomical connections linking neural elements. A representation of the structural connectivity of the brain corresponds to a connectome, or neural wiring diagram (Sporns et al. 2005). There are various available methods for mapping structural connectivity, including postmortem dissections and non-invasive imaging techniques such as structural fMRI and diffusion fMRI (Sporns 2012). *Functional connectivity* refers to patterns of symmetrical statistical association between activities in different neural elements. Measured in terms of correlation or covariance, mutual information, or spectral coherence between activities in neural elements—regardless of whether they are structurally connected—functional connectivity captures neurophysiological dynamics (Friston 1994). A third mode of connectivity is *effective connectivity*, which refers to patterns of causal relations among neural elements. Effective connectivity is measured using methods such as dynamic causal models, graphical models, structural equations models, and describes asymmetric relationships between neurophysiological events (Friston 1994, 2011).

Information about these three modes of brain connectivity grounds a theory of cognitive architecture. Such a theory specifies how the system is physically and functionally organized and how the orchestrated activity of its elements is causally responsible for cognitive phenomena and behavior. Information about structural, functional, and effective connectivity would then help scientists identify the topological, statistical, information-theoretic, and causal principles that lie behind the architecture of a cognitive system such as the human brain. In particular, and coherent with the stated goals of the Human Connectome Project, comprehensive maps of connections within an organism’s nervous system should advance understanding of how causal relations between certain neural elements produce specific kinds of cognitive phenomena (Seung 2012; Sporns 2012; Pessoa 2014; Smith et al. 2015).

However, the interrelationships between structural, functional, and effective connectivity are not well understood, and have received little attention in the philosophy of neuroscience. In particular, it is unclear when the multiple ways by which structural, functional, and effective connectivity are measured lead to coherent conclusions about causality (Horwitz 2003). It is also unclear when and how inference about effective connectivity relies on knowledge of structural connectivity, and on patterns of functional connectivity of different brain components (Sporns 2007: 4.3; Ramsey et al. 2010; Friston 2011). Despite these unclarities, the language of connectivity enables us to formulate more precise questions about how different types of relationships are linked. In particular, we may ask how the map of the structural connections at a spatio-temporal scale relates to the map of effective connections at that same scale. By formulating the question in this way, we move away from vaguer questions about whether the causal relations in the brain depend on its physical properties towards more productive questions about how different forms of connectivity are related.

Recently, there has been increased attention to network theory in the literature on mechanistic explanation (Huneman 2010; Levy and Bechtel 2013; Rathkopf 2015; Craver 2016; Kostic 2016). This literature has not spelled out the relationship between the different forms of connectivity in the brain and how they bear on questions of explanation and discovery. It has focused on whether network models for complex systems enable one to provide a form of “topological explanation” of a system’s behavior in terms of its organization, and on how such explanations differ from mechanistic explanations.

Our project is orthogonal to the issues raised in this literature in two ways. First, we are primarily concerned with *discovery*, rather than explanation. Whether or not causal explanations of the brain provide the primary, or the only form of explanation in neuroscience, we need an account of how causal relationships can be discovered. Second, we take much of the existing literature in philosophy to be primarily concerned with comparisons of models that consider a system at two levels of abstraction. For instance, Levy and Bechtel (2013) and Rathkopf (2015) extol the virtues of network models that abstract away from the localized causal details emphasized by mechanistic explanations. In contrast, by focusing on the three types of connectivity at a single time scale, we aim to compare these distinct network models at a single level of abstraction. To have a complete picture of explanations that appeal to topology and those that appeal to localized causes, we need to know how the localized causal relations can be effectively discovered in the first place.

## Mechanism Discovery and the Structure-Causality Relation

With the conceptual tools we have just introduced, we can now examine what roles findings about the anatomical organization of brain circuits play in mechanism discovery. In this section, we focus on the discovery of the mechanism of long-term potentiation (LTP), which mechanistic philosophers such as Craver and Darden (2001) use to illustrate how mechanism discovery strategies rely on findings about brain anatomy. After providing a more nuanced account of the discovery of LTP, we conjecture that contemporary strategies grounded in network neuroscience will rely less and less on detailed information about structural connectivity.

Mechanistic philosophers have characterized investigative strategies scientists use to discover mechanisms well before the rise of network analyses in neuroscience. Bechtel and Richardson (1993), for example, focus on strategies of localization and decomposition, whereby scientists begin from a description of an explanandum phenomenon, which, during the course of experimental research, gets decomposed into sub-functions that are localized on distinct parts of a mechanism. Darden (2002) and Craver and Darden (2013) discuss how some prominent strategies for mechanism discovery in biology and neuroscience involve constraint-based reasoning to narrow down the space of possible mechanisms for a given phenomenon. Findings about the spatial organization of a putative mechanism provide one relevant constraint. The spatial organization of a mechanism includes the physical connections between its parts. Getting the physical connections right can aid mechanism discovery, because what a mechanism does is constrained by how the entities in the mechanism are physically interconnected.

Mechanisms are said to consist of patterns of dynamic causal relations, including “patterns of allowance, generation, prevention, production, and stimulation” (Craver 2007: 136). These patterns of causal relations are said to be “sustained” or “scaffolded” by the spatial organization of a mechanism in the sense that different structural arrangements of the component parts allow for different patterns of causal relations. As anatomical connectivity is one feature of the spatial organization of brain mechanisms, different patterns of causal activities depend on different patterns of anatomical connectivity. New information about the neural “wiring diagram… alters the space of plausible mechanisms by changing the scaffolding on which the mechanism can be constructed” (Darden 2006: 53).

Craver and Darden (2001) examine the history of the discovery of long-term potentiation (LTP) to illustrate how this constraint-based reasoning strategy often relies on findings about brain anatomy. LTP is a kind of synaptic plasticity that was initially observed and described by the Norwegian neuroscientist Terje Lømo in the 1960s. More specifically, LTP is a persistent increase in the strength of synapses between two neurons following high-frequency stimulation of a synapse. This strengthening leads to an increase in signal transmission between the two neurons. Since changes in synaptic strength are thought to update and store information, LTP is believed to constitute the synaptic basis of the mechanisms of learning and memory.

In discussing Lømo’s work, Darden (2006: 53) points out that early research on the hippocampus assumed that “the anatomical connectivity of hippocampal regions exhibits a characteristic ‘trisynaptic’ loop’” and that this assumption constrained the search for the mechanism of LTP. Darden does not elaborate on how anatomical knowledge constrained or guided Lømo and collaborators’ inferences about the causal organization of the hippocampus and underlying LTP.

Craver (2003, 2007: Ch. 7) provides us with more details. He describes research conducted by Lømo with his collaborator Tim Bliss in the late 1960s and early 1970s. Lømo and Bliss’s (1973) research was motivated by Lømo’s earlier observation that the dentate area of the hippocampus remains potentiated for a significant time after short periods of electrical stimulation of its afferent perforant pathway (Bliss and Lømo 1970; Lømo 1971). As Lømo and Bliss wanted to understand the causes of this phenomenon, they examined the causal relationship between activity in the perforant path, the main input to the hippocampus, and in the dentate gyrus, whose granule cells receive the major excitatory input of the hippocampal formation from the cortex and other areas upstream.

Accordingly, Lømo and Bliss started a number of electrophysiological studies, where they stimulated the presynaptic fibers on the perforant pathway in the brain of anaesthetized rabbits, and recorded responses from populations of neurons of the dentate gyrus. High-frequency electrical stimulation resulted in an input-specific, long-lasting change in synaptic responsiveness to subsequent input of cells of the dentate gyrus. Bliss and Lømo called this effect “long-term potentiation”, and hypothesized LTP as a candidate cellular mechanism for memory (Bliss and Lømo 1970, 1973; Lømo 2003; Andersen et al. 2006).

Craver (2007: Ch 7) makes three points about this case study: (1) Bliss and Lømo’s experiments illustrate the interfield integration of anatomical and electrophysiological results; (2) Bliss, Lømo, and their collaborators used several different techniques to identify the anatomical organization of the hippocampus; and (3) Bliss and Lømo could use the wiring diagram of the hippocampus “as a foundation for electrophysiological investigation” (239). The third point is particularly relevant for our purposes. Craver qualifies it in these terms:

Using the anatomical map of the hippocampus, one could intervene to change the electrophysiological properties of specific cells or populations of cells (for example, by delivering current) and could record the effects of those interventions on other cells or populations of cells. In this way, electrophysiologists could study the propagation of neural excitation through the circuitry of the hippocampus (Ibid.)

Here Craver highlights that anatomical information played an important role in the experimental design of Bliss and Lømo’s studies. By the 1960s, Ramon Y Cajal’s (1911) and Lorente de Nó’s (1934) descriptions of the anatomy of the hippocampus, of its cytological structure and of its structural connections with other brain regions were well-known. The hippocampus was viewed as a simple neural structure, which was accessible for neurophysiological manipulation and could serve as a prototype model for studying general cortical synaptic mechanisms (cf., Lømo 1971).

The anatomy of the hippocampus facilitated electrophysiological studies, where synapses on the perforant pathway were stimulated, and recordings of synaptic signals were taken from the cells in the dentate gyrus. Based on their anatomical knowledge, Bliss and Lømo decided to place stimulating electrodes beneath the angular bundle to activate 35 distinct paths to the dentate area (1973: 333–335). To characterize the effects of electrical stimulation, Bliss and Lømo (1973: 335) considered three parameters: (1) The amplitude of the population excitatory postsynaptic potential; (2) the peak-to-peak amplitude of the population spike; and (3) the latency of the population spike. The effects of electrophysiological stimulation on these parameters showed great variability between subjects, and within the same subject over trials. In particular, some subjects did not show changes in any of the three parameters. Nine of the 35 paths that were stimulated showed long-lasting changes in all three parameters. Reduced latency appeared in more than half of the trials; and one in four trials exhibited changes in at least one of the three parameters 30 min after the stimulation.

While anatomical knowledge clearly played a role in Bliss and Lømo’s (1973) experimental design, it is less clear that Bliss and Lømo relied on such knowledge in causally interpreting their results. Bliss and Lømo’s (1973: 350–351) own account emphasizes that cable theory played a central role in constraining their inferences about the most likely cause of the increase in the amplitude of the synaptic response they observed.

Developed after the work of Wilfrid Rall and of Hodgkin and Huxley in the 1950s and 1960s, cable theory includes mathematical models describing the propagation and interaction of electrical currents in neuronal circuits. Models from cable theory provide a quantitative understanding of how membrane potential changes flow through dendrites, by representing basic properties that causally affect the propagation of synaptic currents, like membrane resistance, membrane capacitance, and intracellular resistance.

Using cable theory, Bliss and Lømo (1973) modeled how changes in the amplitude of an evoked population potential could causally depend on changes in the cable properties of hippocampal neurons. They concluded that a change in synaptic efficacy was the most likely cause of the increase in amplitude of the synaptic response, and that LTP might be a component in a mechanism for memory (cf. Andersen et al. 2006).

Craver is right to say that knowledge of the wiring diagram of the hippocampus served “as a foundation for electrophysiological investigation” (239). Available knowledge of the anatomical organization of the hippocampus was of great help to Bliss and Lømo for designing their experimental studies. They knew which pathways of the hippocampal formation were the most accessible for stimulation with electrodes; and they knew where evoked synaptic responses could be more easily recorded. However, inferences about the causes of LTP and the suggestion that LTP might be involved in memory depended more clearly on Bliss and Lømo’s knowledge of modeling results from cable theory and of statistical relations between recorded neural activities than on their knowledge of the anatomical organization of the hippocampus.

The observation that knowledge of anatomical connectivity did not obviously constrain Bliss and Lømo’s (1973) inferences about the causal organization of the mechanism of LTP coheres with early definitions of the concept of *effective connectivity*, which emerged in the 1970s in the context of single-unit electrophysiology as an attempt to use statistical correlations between spike trains to detect causal interactions between neural activities (Gerstein and Perkel 1969; Moore et al. 1970).

In single-unit electrophysiology, functional connectivity was initially defined as the “temporal coherence” among the spike trains of different neurons, and was measured by cross-correlating neurons’ spike trains. Effective connectivity was defined as the simplest circuit model “that would replicate the experimentally observed features of measurements made on simultaneously recorded spike trains. Thus, when we make the jump from observed coincident spike events to a statement of *effective connectivity* between two neurons, this should be taken as an abbreviated description of an equivalent class of neural circuits” (Aertsen et al. 1989: 900). Structural connectivity plays no role in this definition, which emphasises that hypotheses about effective connectivity should explain dynamical behavior providing links to computational models of brain function.

Interestingly, after more than 40 years since Bliss and Lømo’s initial studies, and with the rise of network analyses, it remains controversial how the pattern of effective connectivity of the LTP relates to hippocampal-dependent memory. To make progress on this outstanding question, Bliss and collaborators have recently proposed “a shift from mechanistic investigations of synaptic plasticity in single neurons towards an analysis of how networks of neurons encode and represent memory” (Neves et al. 2008: 65). Such an analysis—they suggest—would be focused on cross-correlations of activity in distinct neural circuits on the hippocampus. The aim is to identify increases (or decreases) in the peak of the cross-correlogram, which can then be used to infer changes in the strengths of the synaptic coupling between target circuits after experimental manipulation. This proposal indicates that future inquiry into the causal basis for LTP may well move away from detailed examinations of structural connectivity, rather than towards it.

## Structure, Statistics, and Effective Connectivity

Now that we have situated our discussion in the broader context of mechanism discovery, we turn to the question of whether findings about structural connectivity constrain the space of possible hypotheses about effective connectivity in current quantitative approaches for causal discovery in neuroscience.

Some of these approaches, by which one seeks to discover the causal organization of the brain, look different from the experimental methods that have been described in the earlier literature (Bechtel and Richardson 1993; Darden 2002; Craver and Darden 2013). Even for models with as few as five nodes representing regions of interest, there are tens of thousands of possible causal models representing the relationships among the nodes (Glymour and Hanson 2016), and it is typically infeasible to uniquely identify the correct causal model. Consequently, finding a plausible model is not just a matter of strategically performing experiments, but further involves developing principles for narrowing down the set of causal models that are deemed to be best supported by the evidence. We refer to this process of narrowing down the set of considered models as *causal inference*. Insofar as causal inference relies on epistemic principles for selecting among alternative causal models, it involves rational principles for discovery. Of course, even the LTP studies just described involved inferences regarding patterns of effective connectivity. Nevertheless, the increased complexity of network representations calls for a more systematic treatment of the epistemic principles for causal inference.

One route for evaluating how structural knowledge constrains causal inference in neuroscience begins with the observation that knowledge of statistical relationships can justify causal claims only in conjunction with additional assumptions. Now, if these assumptions concerned structural connections among brain elements, then one could not use statistical relationships to establish causal claims without relying on knowledge about the anatomical structure of the brain. But do these assumptions actually refer to structural connections? Here we consider this question in the context of contemporary methods for causally modeling the brain, starting with Dynamic Causal Models (DCMs).

The use of DCMs is one of the most prominent approaches to causal inference from neuroimaging data (Friston et al. 2003). DCMs allow one to measure the patterns of causal interaction among brain regions and to predict how these relationships will change over time in response to experimental interventions. These models include not only a representation of the causal relationships among brain regions, but also of the biophysical processes by which the changes in brain activity produce one’s data. Accordingly, DCMs for fMRI involve a hemodynamic model for how changes in the level of blood oxygen in the brain produce the BOLD (blood oxygenation level dependent) signal. Using Bayesian model selection procedures, it is possible to use fMRI data to make inferences about the magnitudes of the interactions among regions.

Dynamic causal modeling involves two main steps (Valdes-Sosa et al. 2011). The first consists in specifying a set of equations corresponding to the causal interactions in the brain and the manner in which they produce the data, and the second consists in statistically estimating the causal parameters in those equations. In the first step, state-space models of effective connectivity of a target neural system are specified. This requires the definition of a set of neural variables, which are associated with brain components at different spatial scales, most commonly to whole brain areas. It also involves the definition of two sets of mathematical models of bio-physiological processes in the brain, which specify how the neural variables change over time, and how the hidden states of the neural variables generate observable data. One set of mathematical models uses differential equations to represent the dynamics of hidden neurophysiological states, like synaptic activity, corresponding to the nodes of a probabilistic graphical model, where conditional dependencies are parameterised in terms of directed effective connectivity couplings. These linear parameters pick out the direction and strength of possible causal couplings between different hidden states of a graphical model, and the goal of DCM is to estimate these “effective connectivity parameters”.

As the states of the neural variables are hidden (i.e., not observed or measured directly), a set of equations is also defined to represent how the BOLD response reflected in the fMRI time-series data is generated from the unobserved, underlying neural dynamics. In formulas, DCMs are characterized by two sets of equations: state equations (Eq. 1), and observation equations (or hemodynamic models) (Eq. 2):

1 $$\frac{\partial x}{\partial t} = f\left( {x,u,\theta } \right) + w$$

2 $$y = g\left( {x,u,\theta } \right) + v$$

Together, the sets of equations corresponding (1) and (2) provide a DCM. In these equations, *x*s denote neural variables, *u*s denote exogenous inputs typically corresponding to experimental interventions, and *θ*s denote the coupling parameters picking out the strengths of the causal connections between variables. Equations of form (1) indicate how the coupling relationships among variables change in response to interventions. Equations of form (2) indicate the hemodynamic mechanisms for how the BOLD signal is produced by the underlying activities given by (1). Of course, realistic DCMs will contain a large number of variables and equations. The parameters in these equations may be organized using matrices.

Given (1) and (2), the second step involves model inversion and estimation. Competing models of neural causal dynamics can be compared on the basis of observed time-series data using model selection methods that balance model fit and complexity. The aim is to estimate which set of parameter values for (1) is most likely to have produced the observed fMRI data. In DCM, it is common to use Bayesian model selection methods, where one assigns a prior probability distribution over the possible parameter values in a model and then conditionalizes on the data to update one’s parameter estimates. The dynamical model with the highest posterior probability from the set of models under consideration can thus be used to estimate coupling parameters that specify the causal relations between different states in the associated graph.

There are several ways that DCMs might be thought to incorporate anatomical information. First, one might suppose that what makes these models causal (as opposed to merely statistical) models is that they represent the anatomical relations among brain regions. While there are philosophers who explicate causal relationships in terms of physical connections (Salmon 1984; Dowe 2000; see also Handfield et al. 2008), these metaphysical accounts are controversial, and in any case unnecessary for distinguishing causal from merely statistical models. Causal models can be satisfactorily distinguished from merely statistical ones on the grounds that only the former allow scientists to reliably predict the results of interventions.

The temptation to treat DCMs as essentially referring to anatomical structure is strengthened by an equivocation in the term ‘structure’. The relationships among variables in approaches like DCM are sometimes referred to as ‘structural’ in the sense that the distinct equations may be independently disrupted via an intervention. Clearly, the claim that a model is structural in this sense does not entail anything about its relationship to anatomical structure.

A second way in which DCMs may incorporate anatomical information is through the ‘observer’ or hemodynamic models of how the fMRI time-series data is produced by the underlying neuronal activity. Although these hemodynamic models are essential for learning causal models from the data, they appear to play no role in distinguishing among different causal models at one level of grain. One cannot, for example, use the hemodynamic model for distinguishing among different hypotheses regarding which brain regions are causally related by coupling parameters. After all, the hemodynamic model does not represent any relationship between neural states. What it does is assume that neural activity induces a vasodilatory signal that increases blood flow, which, in turn, leads to changes in blood volume and deoxyhemoglobin content. As a function of these latter two hemodynamic states, the hemodynamic model outputs a predicted BOLD response. In other words, the hemodynamic model specifies how the underlying states produce the data, rather than how individual states causally relate to one another.

The third and most plausible proposal for how DCMs incorporate anatomical facts is that knowledge of structure can be helpful to narrow down the set of candidate causal hypotheses associated with a DCM. But what evidence is there that structural information actually informs neuroscientists’ inferences regarding effective connectivity? And in what ways does knowledge of structure constrain the set of candidate causal hypotheses?

**Fig. 1:**
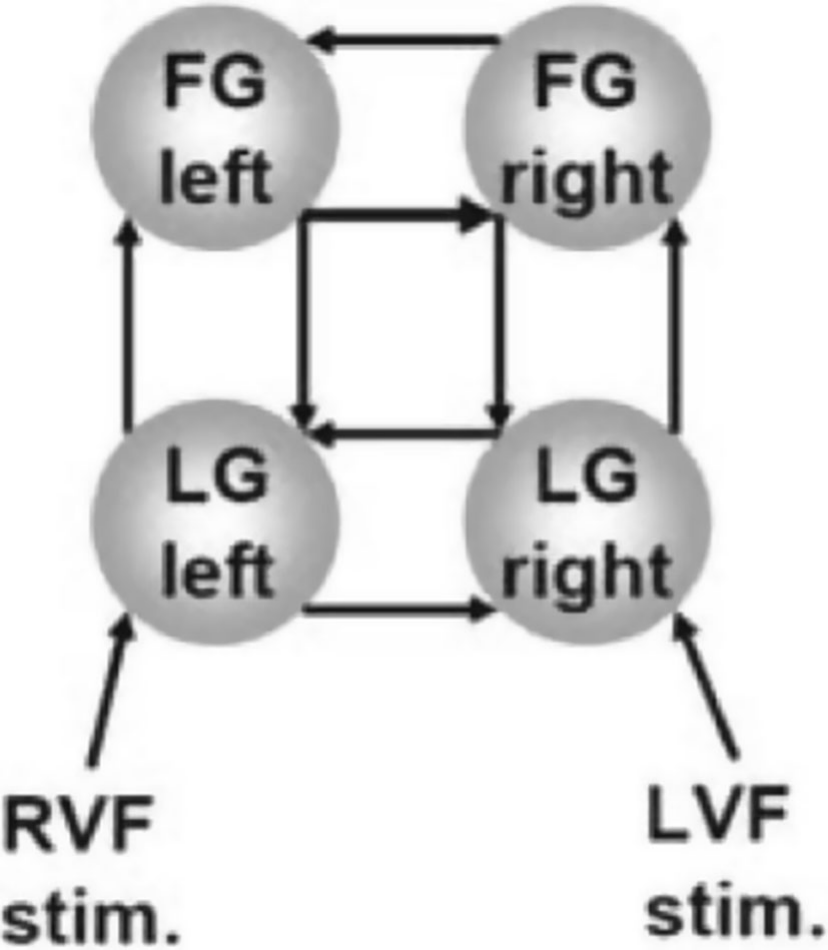
Graph from Stephan et al. (2007). *FG* fusiform gyrus, *LG* lingual gyrus, *RVF/LVF* *stim* right/left visual field stimulation

After noting how there is only limited evidence that knowledge of structure plays a role in causal inference from fMRI data, Friston (2011: 16) directs us to Stephan et al. (2009), which claims to provide “the first formal demonstration that knowing anatomical connectivity improves inference about effective connectivity” (1635). Stephan et al. (2009) rely on a graphical model for intra-hemispheric visual processing from Stephan et al. (2007). The model represents a system including four nodes corresponding to distinct brain regions: the lingual gyri (LGs) and the fusiform gyri (FGs) for the left and right hemispheres. The model contains reciprocal connections between the LGs and FGs in each hemisphere and between the left and right LGs and the left and right FGs (see Fig. 1).

Information about anatomical at a spatial and temporal scale useful to constrain DCM is derived from tractography and is given in terms of the probability that there is an anatomical link between two given regions. Stephan et al. (2009) reason that the more probable it is that there is an anatomical connection between two variables, the lower the variance is in the prior probability distribution over the values of the coupling parameters between variables in the state equations they consider. The distributions over these parameters have mean zero, and having lower variance makes it more likely that they will have non-zero values in their posterior distribution. In other words, having a lower variance makes it more probable that one will find a causal connection (whether positive or negative) if there is one. Knowledge of anatomical structure was used to assign prior probabilities to the effective connectivity parameters in the set of DCMs under consideration.

This strikes us as one sensible way of using anatomical information to constrain causal inference. After all, if there is no anatomical path connecting two regions, then those two regions cannot causally interact. But what was the exact role of these anatomically-informed priors in causal inference?

Stephan et al. (2009) used a Bayesian model selection method to estimate coupling parameters between four ROIs. Anatomical information was used to set the prior distributions of these parameters. One could either make the variance of the priors inversely proportional to the probability of a connection, as Stephan and colleagues did, or (counterintuitively) make it proportional to the probability of a connection. Within each of these options, one can vary the degree to which the probability of an anatomical connection influences the variance in the prior distribution. One could also use a flat prior distribution that does not incorporate information about structural connectivity.

Stephan and colleagues generated 30 sets of parameter values assuming an inverse relationship between connection probability and variance, 29 sets assuming these are proportional and 3 sets of parameters for the flat distribution. They then asked: What is the optimal model? That is, what is the model that maximizes the marginal likelihood of the coupling parameters?

To answer this question, they used a measure of model evidence that balanced accuracy (parameters log-likelihood) with model complexity (number of parameters). Specifically, Stephan and colleagues used a model selection method based on the free energy of different models. Thus, they evaluated the probability that each parameterized model produced the data. Of the ten top models, 7 of them—including the top 6—relied on anatomically informed priors in which the prior distribution was inversely proportional to the probability of a structural connection.

Our key concern with the reliability of this approach for causal discovery is that it is unclear why we should take the models that score highest to reflect the true causal models. The authors emphasize that Bayesian model selection methods do not merely compare models in terms of which best fits the data, but balances fit against complexity to avoid over-fitting. But relying on more sophisticated statistical methods does not by itself ensure that one will get the correct causal model. Variables that do not cause one another may nevertheless be good predictors of one another, for example if they are the effects of a common cause. Without explicit causal assumptions, Bayesian model selection methods cannot distinguish between variables that cause one another and those that merely predict one another.

In fact, the sets of models that Stephan et al. (2009) considered did incorporate causal assumptions. First, the state-space of models of interhemispheric integration they considered had been previously evaluated in experimental contexts, enabling one to eliminate confounding (cf. Stephan et al. 2007). Furthermore, the set of state equations considered by Stephan et al. (2009) assumed that there are no direct causal relationships between variables that are not connected by an arrow, such as FG left and LG right.

Of course, to say that these authors relied on causal assumptions is not to say that the models they produce are problematic. Yet, their discussion obscures the role that these assumptions play in causal inference. The authors’ causal assumptions played the role of limiting the set of models to which they applied their Bayesian model selection techniques. These model selection techniques incorporate information about structural connectivity in terms of priors over coupling parameters, but the role played by this information is to allow for more efficient statistical estimation of the effective connectivity parameters. Stephan et al’s (2009) study, therefore, illustrates that anatomical findings facilitate causal discovery in DCM by aiding statistical inference of effective connectivity parameters in a predetermined set of causal models.

We need to further clarify the sharp line we are drawing here between inferring the correct causal model and statistically estimating the parameters of that model. After all, in providing improved ways to estimate the parameters in a DCM, Stephan et al. (2009) are not *just* doing statistical inference, since the quantity estimated is a causal parameter. Yet we have claimed that their methods in no way test their models’ distinctively causal assumptions. To clarify this point, we will utilize the probabilistic causal models of Pearl (2009) and Spirtes et al. (2000). In addition to providing clarification of the distinction between causal and statistical inference, these models have been used specifically for causally modeling the brain (Hanson et al. 2013), and they do so without relying on findings about structural connectivity. Accordingly, a comparison between probabilistic causal models and DCMs will illuminate whether information about structural connectivity is indispensable for reliable causal discovery.

Pearl (2009) and Spirtes et al. (2000) represent causal hypotheses using directed acyclic graphs (DAGs), in which the nodes are random variables, and the edges represent direct causal relationships among the variables. “Directed” indicates that all edges in the model are asymmetric, and “acyclic” indicates that there are no causal cycles. The acyclicity assumption may be dropped, yielding a directed *cyclic* graph (DCG). The properties of cyclic graphs are well understood (Park and Raskutti 2016), though significantly more complicated than those of DAGs. We will therefore presently focus on the latter.

It is useful to refer the relationships among variables in a model by analogy to genealogical relations. A variable’s direct causes are its *parents* and its effects (whether direct or indirect) are its *descendants.* DAGs are associated with probability distributions, and the condition of adequacy for a DAG to be compatible with a probability distribution is given by the *Causal Markov Condition:*

*Causal Markov Condition* (CMC): Each variable is a causal model is probabilistically independent of all of its non-descendants, conditional on its parents.

Intuitively, the CMC captures the idea that a variable and its descendants are only connected to other variables in a model *through* its parents, so once one knows the values of its parents learning the values of other non-descendants will not help one better predict its value.

Given the CMC and a DAG, one can determine which (sets of) variables will be probabilistically independent conditional on which (other sets) of variables. For short, we can refer to these as the conditional independencies entailed by a DAG. Note that whether a probability distribution is compatible with a DAG depends only on the conditional independencies, and not on any other features of the distribution such at the magnitudes of the correlations. Moreover, the conditional independencies entailed by a DAG according to the CMC result from the edges that are *missing* from a graph. A graph with no missing edges—i.e. a complete graph in which every node is directly linked to every other—does not entail any conditional independencies. For this reason, the CMC is often supplemented with additional principles to narrow down the set of acceptable models.

For example, the Causal Faithfulness Condition states that one should not only eliminate models that entail conditional independencies that are not found in the distribution, but that one should also only choose models that entail *all* of the conditional independencies that obtain. In other words, every independency must be entailed by the model (according to the CMC). The Causal Faithfulness Condition, in requiring that *all* independencies in the distribution be entailed by the model, is fairly strong, to the point that there may be *no* model satisfying the condition. Fortunately, there are other, logically weaker, supplements to the CMC that are available (Zhang 2012; Forster et al. 2017) though the differences among them will not concern us here.

DAGs are members of the same *Markov Equivalence Class* (MEC) if they entail all and only the same conditional independencies according to CMC. DAGs in the same MEC are observationally equivalent in the sense that one cannot use the conditional independencies alone to distinguish among different models in the same class.

Glymour and Hanson (2016) describe one way of using the notion of an MEC to combine DAGs with Bayesian model selection methods. They consider Hanson et al. (2013) imaging study on action understanding in individuals with autism, and ask: How can a collection of BOLD time series, one for each (aggregate of) voxel(s), provide evidence about the causal network among brain regions involved in a given experimental task?

In the Hanson study, there were five regions of interest selected on the basis of hypotheses about the areas involved in their experimental task. Thus, 29,281 distinct DAGs were considered, each one of which represented a different possible causal model with five variables. Each graph was associated to a statistical model with free parameters, and a maximum likelihood estimate of the parameters could be obtained.

To identify the best fitting causal model, Hanson and colleagues used a search procedure called ‘greedy equivalence search’ that begins with the MEC of DAGs with no edges. At each step one considers all single directed edges that could be added to each member of the MEC, and evaluates the resulting models based on the Bayesian information criterion (BIC) score, which is a criterion for model selection. Specifically, the greedy equivalence search procedure chooses the MEC that most improves the BIC score. When no further addition of edges can improve the BIC score, the procedure eliminates edges until the BIC score cannot be improved. This search procedure found the causal model that best explained Hanson and colleagues’ data, but did not compare all 29,281 distinct DAGs.

The key is that when comparing models using the search methods used by Hanson et al. (2013) and discussed in Glymour and Hanson (2016), one only needs to consider a single model from each MEC. Without making a priori assumptions about the functional relationship between a variable and its causes, there is no way to use probabilistic information to further distinguish among models within an MEC (see Spirtes and Zhang 2016: 13ff). While this method is compatible with that of Stephan et al. (2009), it neatly distinguishes between causal and statistical inference. Causal inference involves finding the correct causal model, which may be determined up to MEC based on knowledge of conditional probabilities, and possibly further specified using additional information. Using DAGs, it is then possible to determine whether a causal quantity is in principle identifiable from a probability distribution and to derive the probabilistic expression for this quantity (Pearl 2009).

Friston (2011: 25, see also Valdes-Sosa et al. 2011) notes two ways that DCM’s are allegedly superior to DAGs. First, DCMs, unlike DAGs, allow for causal cycles. Yet, as already noted, the DAG framework may be generalized to allow for cycles. Second, DAGs do not incorporate time-ordering information. This is often the case, though there is no reason that DAGs cannot be supplemented with time-ordering information. Such information is perhaps the most common way of distinguishing among DAGs in the same Markov Equivalence Class. What DAGs do is allow one to read off the consequences of a qualitative causal hypothesis. But while it is common to read off time ordering from causal ordering, most philosophers hold that it is not part of the definition of causation that causes precede their effects.

To be clear, we do not intend to minimize the methodological issues related to causal inference on systems with complex temporal relations. Causal inference using time-series data poses its own challenges, which we do not have space to address here. As with spatial relations, it will be important to get clear on the particular role played by time in causal inference.

Within the DAG framework, functional and effective connectivity are more closely related than one would expect from reading the literature on DCMs. While one cannot infer effective connectivity from functional connectivity, it is nevertheless true that given a causal hypothesis, one can take the relevant functional relationships to provide measures of causal strength.

Friston sometimes illustrates the distinction between functional and effective connectivity by considering functional relationships that are used for the purely predictive task of categorizing subjects into different classes (Friston 2011: 15). This usage does not seek to establish causal relationships among the variables, but to distinguish between the different types of individuals from which different data were sampled. If one *were* to interpret these methods as providing causal information, it would be in terms of the causal relationship between a latent variable and the measured variables. It is clear enough that one should not use the functional relationships measured in these methods to draw causal inferences about the variables in one’s model. But one should not use this point to draw a more general lesson about the relationship between functional and effective connectivity. Here we have been concerned with how functional connections among measured variables relate to effective connections among those *same* variables. Given the causal assumptions embedded in a DAG, one can sometimes use the strengths of functional relationships to learn about those of causal ones.

In summary, in DCM information about structural connectivity has been used to improve one’s ability to efficiently estimate the parameters in a model. Yet, DCM does not transparently incorporate epistemic bridge principles that connect fMRI data to the underlying causal structure. If the sets of differential equations describing neuronal and physiological dynamics in DCM do not correspond to equations that may be interpreted causally, then Bayesian model selection will not deliver causal knowledge. Probabilistic graphical methods do a better job at distinguishing between causal and statistical inference. However, even in the ideal case, where one has knowledge of all conditional independence relations between the measured variables of a target brain system, one can do no better than discovering the correct Markov Equivalence Class. It is an open and underexplored question whether (and how) connectomic information is required to pick out a particular causal model within a Markov equivalence class as the true causal model.

## Conclusion: Modes of Connectivity, Discovery and Explanation

In this paper, we asked how findings about anatomical connections in the brain contribute to causal inference in neuroscience. We situated our discussion in the literature on mechanistic discovery to evaluate the role of anatomical information in causal discovery more generally. In electrophysiological studies like Bliss and Lømo’s (1970, 1973), anatomical findings played an important role in the design of experiments and in picking out possible constitutively relevant components of the mechanism of a given phenomenon. However, anatomical information did not play an evident role in the interpretation of the experimental results about LTP. We subsequently considered the alleged role of anatomical information in interpreting the results of functional imaging techniques. On the basis of an examination of causal modeling approaches, we showed that information about anatomical connections does not currently play a substantial role in causal inference in neuroscience.

Our discussion makes several contributions. First, we have clarified the relationships between structural, functional, and effective connectivity. A reader of the literature on dynamic causal modeling would be left with the false impression that one of the main advantages of these models is in allowing neuroscientists to incorporate anatomical information into their causal inferences. We have sought to correct this impression and to also illuminate ways in which statistical information plays more of a role in guiding causal inference than is often attributed to it. More generally, we have pinpointed what it would mean for anatomical information to contribute to causal, as opposed to statistical inference, in these modeling methods.

Second, we fill a gap in the mechanistic literature, which began discussing causal discovery before the advent of network neuroscience and powerful causal modeling techniques, and has recently considered the implications of network theory for explanation. Here we have argued that causal discovery poses particular challenges in the context of network neuroscience. The relationships among anatomical, statistical, and causal facts looks markedly different from the perspective of discovery than they do when discussing explanation. Whereas in mechanistic approaches to both explanation and discovery anatomical information, but not statistical information, is critical, in network approaches to causal inference, in contrast, statistical information plays an essential role, while anatomical information does not.

Our discussion points towards several directions for future research. We have not addressed the new and exciting questions that have arisen regarding the explanatory import of networks, and in particular have not taken a stance on whether there are forms of topological explanation that abstract away from the relevant causal-mechanical details. Surely this debate will benefit from a clearer picture of which causal relationships exist in the brain, and how neuroscientists attempt to discover them. Additionally, we have limited ourselves to considering systems represented at a particular time-scale, though to adequately understand brain function and behavior we will need to evaluate its neurodynamics at multiple scales. For instance, we would want to know how the stability of brain function at a longer time scale depends on its anatomical and causal organization at shorter time scales. While there remains much more work to be done, the present discussion showcases why network measures will be at the forefront of future developments in causal inference and mechanism discovery in the brain sciences.
